# Gene therapy for inborn errors of liver metabolism: progress towards clinical applications

**DOI:** 10.1186/1824-7288-34-2

**Published:** 2008-11-18

**Authors:** Nicola Brunetti-Pierri

**Affiliations:** 1 Department of Molecular and Human Genetics, Baylor College of Medicine, Houston, TX, USA

## Abstract

The treatment for inborn errors of liver metabolism is based on dietary, drug, and cell therapies (orthotopic liver transplantation). However, significant morbidity and mortality still remain, and alternative strategies are needed. Gene replacement therapy has the potential of providing a definitive cure for patients with these diseases. Significant progress has been made in the pre-clinical arena and achievement of efficacy in different animal models has been reported using multiple gene transfer technologies. This article summarizes the gene transfer strategies being investigated, the pre-clinical data, and the available early clinical results for inborn errors of liver metabolism.

## Introduction

Significant advances in the diagnosis and treatment of inborn errors of metabolism have occurred in recent years. Expanded newborn screening using tandem mass spectrometry has led to the ability to identify and treat neonates who have metabolic conditions before symptoms appear [1]. Developments in nutritional support and pharmacological treatments with vitamin cofactors, end-product replacement, and drugs inducing specific enzymes or alternative pathways have also led to better outcomes. Cell therapy, primarily orthotopic liver transplantation (OLT), has significantly changed the prognosis of some of these diseases. Patients undergoing solid organ transplantation have benefited from innovative surgical techniques and novel, less toxic nonsteroidal immunosuppressive regimens. However, pharmacological treatments are often insufficient in the face of the activation of catabolic states, many patients succumb while waiting for a donor organ (approximately 15%), and short-term peri-transplant morbidity and long-term morbidity associated with lifelong immunosuppression continue to be significant issues [2-5]. Therefore, a risk/benefit assessment could make gene therapy an acceptable option for several inborn errors of metabolism.

Progress in the direction of clinical application of gene replacement therapy has been scarce so far despite extensive investigations for over 20 years. A general skepticism toward gene therapy was raised by the death of one patient in the ornithine transcarbamylase deficiency (OTCD) clinical trial [6] and by the recent report of leukemia occurred in few patients with severe combined immunodeficiencies (SCID) treated with retroviral *ex vivo *gene therapy [7]. However, with regard to the SCID trial, it is important to emphasize that despite the adverse events, it clearly demonstrated the benefits of gene therapy as treated patients can now cope with environmental microorganisms and live a normal life in the absence of any specific therapy [8].

Several different types of vectors, both viral and nonviral, have been developed for liver-directed gene therapy and have resulted in phenotypic correction in numerous animal disease models. The optimal vector for *in vivo *liver-directed gene therapy should be able to transfer genes to a high percentage of hepatocytes with limited toxicity. However, the available vectors have all shown some limitations (Table 1). In aiming at the treatment of liver metabolic diseases an important issue is how much of the liver needs to be corrected (i.e. percentage of hepatocyte) to achieve clinically relevant improvements. The percentage of hepatocyte transduction required for phenotypic correction is generally low in non-cell autonomous disorders such as hemophilia A and B or mucopolysaccharidoses and higher in cell autonomous defects such as urea cycle disorders. As a general principle, maximizing therapeutic gene expression per cell and minimizing the vector dose for a clinical effect are desirable. However, the potential adverse effects of over-expression of the therapeutic protein should also be taken into account.

**Table 1:** Overview of gene therapy vectors.

	***Genetic material***	***Packaging capacity***	***Vector genome forms***	***Advantages***	***Disadvantages***
**Retrovirus**	RNA	8 kb	Integrated	- High efficiency integration	- Transduction only in dividing cells
				- No viral immune response	- Insertional carcinogenesis
				- Long-term expression	
**Lentivirus**	RNA	8 kb	Integrated	- Non-dividing cells	- Integration into active genes
				- Long-term expression	- Risk of replication competent HIV
**Adenovirus**	dsDNA	Up to 35 kb (HDAd)	Episomal	- Non-dividing cells	- Acute toxicity
				- Large cloning capacity	
				- High transduction levels	
				- Long-term expression (HDAd)	
**Adeno-associated vectors**	ssDNA	5–9 kb	Episomal (> 90%)	- Non-dividing cells	- Limited cloning capacity
			Integrated (< 10%)	- Long-term expression	- CTL-mediated immune reaction
**Naked plasmid DNA**	dsDNA	Unlimited	Episomal	- Non dividing cells	- Low efficiency of transduction
				- No inflammatory response	- Efficient and clinically relevant delivery method still to be developed
				- Large cloning capacity	
				- Long-term expression	
				- Ease preparation	

dsDNA = double stranded DNA; ssDNA = single stranded DNA; HDAd = helper-dependent adenoviral vector; CTL = cytotoxic T lymphocyte.

### Vectors for liver directed gene therapy

The number of different vectors that are under development for liver-directed gene therapy is continuously increasing. However, five main classes of vectors have been more extensively investigated and each of these classes is characterized by different strengths and weaknesses (Table 1).

#### Retrovirus

Retroviral vectors (RV) were the first vectors used for gene therapy. They can efficiently integrate into the chromatin of target cells. However, they require the target cells to be mitotically active for an efficient transduction. Therefore, induction of liver division or liver regeneration through manipulations such as partial hepatectomy or hapatocyte growth factor treatment have been required for efficient hepatocyte transduction [9-11]. More recently, it has been shown that RV can transduce hepatocytes from newborn mice [12] and dogs [13] without an exogenous stimulation of cell division. However, as shown by the SCID trial experience, the risk of insertional mutagenesis is still a major consideration for RV.

#### Lentivirus

Lentivirus vectors (LV) offer similar advantages to the RV, in that they mediate long-term integration of the therapeutic transgene, but unlike RV, they do not require cellular mitosis to gain access to the host genome for integration. They also are thought to share the potential for insertional mutagenesis with subsequent carcinogenesis, although this has not been observed yet in animal models. Following systemic LV delivery, the majority of transduced liver cells are of nonparenchymal origin and therefore, the efficiency of hepatocyte transduction is relatively low.

#### Adenovirus

Adenovirus (Ad) vectors are well suited for liver-directed gene therapy because they can transduce hepatocytes with high efficiency. When tested *in vivo*, first generation of Ad (FGAd) vectors, which are replication-defective but can still express viral genes at low levels, cause acute and chronic toxicity. Helper-dependent adenoviral (HDAd) vectors, which are devoid of all viral genes, offer a better safety profile and can provide long-term transgene expression with negligible chronic toxicity [14]. Several preclinical studies have shown that HDAd results in long-term phenotypic correction in several genetic diseases [14]. However, similar to FGAd they can still cause an acute toxic reaction due to activation of the host innate immune system when they are administered at high dose systemically [15]. A clinical trial for OTCD using an early generation Ad vector bearing the human OTC gene was interrupted when the second subject at the highest dose suffered fatal complications. The trial involved 18 subjects divided into 6 cohorts of 1/2 log dose escalations between cohorts until two subjects were enrolled at the highest dose [16]. As predicted from preclinical models, clinical findings were mild and transient in the initial 17 patients [16]. However, unlike the previous subjects, the last patient enrolled developed within 24 hours after vector infusion a lethal reaction characterized by acute respiratory distress syndrome, hepatitis, disseminated intravascular coagulopathy, hyperammonemia, and high levels of serum IL-6 [6]. Several mechanisms have been proposed to be responsible for the activation of this acute response [17]. However, regardless of the multiple mechanisms involved, systemic administration of Ad results in an acute toxic reaction which is triggered by the Ad capsid proteins in a dose-dependent fashion [15]. This acute toxicity is currently the main obstacle preventing clinical application of HDAd and strategies to overcome this problem are currently under investigation [18-20].

#### Adeno-associated virus (AAV)

AAV vectors are derived from a non-pathogenic human parvovirus that can infect non-dividing cells and remains latent for prolonged periods, predominantly in an episomal state. AAV vectors appear to persist in infected cells and do not trigger a robust innate response following *in vivo *administration. A wide repertoire of different AAV serotypes with different tissue tropisms is now available for several disease applications [21]. AAV vectors have a limited packaging capacity which precludes applications in diseases requiring large therapeutic genes. However, novel AAV serotypes with larger cloning capacity are emerging and they may at least in part overcome this problem [22]. In the clinical study for liver-directed gene therapy of hemophilia B, a recombinant AAV vector expressing human Factor IX (FIX) was infused through the hepatic artery in subjects with severe hemophilia B in an open label, dose-escalation study. Two subjects in the higher dose cohorts achieved measurable FIX levels at 2 weeks after vector infusion but, in contrast to the results generated in animal models, they exhibited a gradual decline in factor levels to < 1% by 10 weeks after vector infusion. This was accompanied in both subjects by an asymptomatic transaminase elevation beginning 4 weeks after vector infusion, with a gradual decline to baseline normal levels coinciding with the loss of FIX expression [23]. This reaction is due to the rejection of transduced hepatocytes by AAV capsid-specific memory CD8(+) T cells reactivated by AAV [24] and intense investigations are currently ongoing to overcome this problem. Another problem of the AAV vectors is as yet theoretical risk of insertional mutagenesis in humans. Studies in mice suggest that AAV vectors are predominantly nonintegrating [25], and a wealth of experience in the field had failed to uncover any evidence of tumor formation as a result of AAV transduction, except for a mouse disease model of mucopolysaccharidosis type VII [26]. A recent study has reported that, in the tumor tissue, the vector appeared to have integrated in a region rich in microRNA sequences on mouse chromosome 12 [27]. On the other hand, follow-up periods ranging up to 9 years in several hemophilic dogs have failed to reveal any evidence of tumor formation [28].

#### Nonviral vectors

Nonviral vectors offer a number of advantages over viral-based strategies, including minimal toxicity from the vector, long-term transgene expression, lack of a humoral response against the vector, and the consequent ability to repeat dose [29]. A major advance in the field has been the development of the hydrodynamic injection technique which involves, in mice, the rapid injection of a large volume of naked plasmid DNA (pDNA) and typically results in 10–15% of hepatocyte transfection [30,31]. Systemic hydrodynamic procedure as practiced in the rodents is neither safe nor practical in larger animals or humans. However, clinically relevant methods using balloon catheters for regional hydrodynamic delivery of pDNA have been developed [32,33]. These studies demonstrate the feasibility of intravascular delivery to the liver using minimally invasive approaches, and are a step in the direction of human clinical trials.

### Pre-clinical and clinical studies

Experimental gene therapy has been used to correct several metabolic diseases. We will discuss two diseases (Crigler-Najjar syndrome type I and OTCD) as representative examples to illustrate the potential and the limitations of currently available strategies for liver-directed gene therapy.

#### Crigler-Najjar syndrome

Crigler-Najjar syndrome is an autosomal recessive condition characterized by non-hemolytic unconjugated hyperbilirubinaemia due to mutations bilirubin-uridinediphosphoglucuronate glucuronosyltransferase (UGT1A1). Patients with Crigler-Najjar syndrome type I (MIM 218800) are refractory to phenobarbital treatment, have life-threatening elevations of bilirubin, and are generally managed with phototherapy throughout childhood and adolescence. Although effective, phototherapy is cumbersome, inconvenient, and its efficacy may diminish with age because of increased skin thickness and decreased surface/mass ratio. Moreover, despite this treatment, patients remain at risk of brain damage when intercurrent infections may increase production of bilirubin above that which can be controlled by the phototherapy [34]. Therefore, patients with Crigler-Najjar type I are often advised to consider liver transplantation, most frequently in the range of 18 – 25 years of age. Crigler-Najjar syndrome has long been considered a paradigm for developing gene therapies for metabolic liver diseases for several reasons: (*a*) the underlying defect is well characterized at the biochemical and molecular level; (*b*) the fraction of corrected hepatocytes required for clinical benefit is small, as deduced from hepatocyte transplantation studies [35]; (*c*) the UGT1A1 does not require strict gene regulation for normal activity; (*d*) an animal model, the Gunn rat, recapitulating the human disease is available; (*e*) the outcome of the experimental therapies can be easily determined by measuring bilirubin fractions in serum and bile; (*f*) the UGT1A1 can be produced from skeletal muscle other than liver, its natural production site, and still retain the ability to transform bilirubin into water-soluble derivatives [36]. For these several reasons, Crigler-Najjar syndrome type I is very attractive as a gene therapy disease candidate and its correction has been the goal of several studies using different vector systems including RV, LV, Ad, AAV, and nonviral vectors. RV expressing UGT1A1 injected in newborns [37] or in conjunction with partial hepatectomy [38] have achieved long-term correction of the hyperbilirubinemia in the Gunn rats. As previously discussed, LV can also transduce nonproliferating cells and, in the Gunn rats, they resulted in stable reduction of bilirubin levels to near normal levels for over 1 year after treatment [39]. Impressive lifelong correction of hyperbilirubinemia has been also reported in the Gunn rats following a single intravenous injection of HDAd vector encoding UGT1A1 with negligible chronic toxicity [40]. Among different serotypes, AAV serotype 1 was found to be the most efficient in correcting the hyperbilirubinemia of the Gunn rats although large hepatic macroscopic lipid lesions of unclear etiology were found in AAV-treated animals [41]. A reduction of hyperbilirubinemia has also been reported following hydrodynamic injection of pDNA [42]. However, as discussed in previous sections, each of the vectors used in this disease model has some limitations which are currently preventing clinical applications.

#### Urea cycle disorders

Urea cycle disorders typically present in the first few days after birth with poor feeding, vomiting, lethargy, and coma due to hyperammonemia. Despite aggressive pharmacotherapy, patients are at high risk for repeated episodes of hyperammonemia and cumulative neurological morbidity and mortality [43,44]. Given these significant problems, gene-replacement therapy could represent a viable alternative to OLT for long-term correction. Several studies over the past decade have found the therapeutic effect of several different FGAd vectors to be transient in the OTCD mouse models and lasting no longer than 2 months [45]. HDAd instead can mediate long-term correction of the OTCD animal model without chronic toxicity [46,47]. The novel AAV serotypes (AAV7, 8, 9), with higher efficiency of hepatocyte transduction, have also resulted in long-term phenotypic correction [48]. The application of LV and nonviral vectors for OTCD has not been reported to date and these vectors are likely to be inefficient in these diseases due to the high percentage of hepatocyte correction required.

## Conclusion

Gene therapy for liver metabolic diseases as an alternative or adjunctive treatment to cell therapy is a logical target given the problems with the available treatment modalities. Disorders such as Crigler-Najjar syndrome and urea cycle disorders are excellent candidates because of their poor prognosis. However, each of the available vector transfer technologies offers strengths and weaknesses. Integrating vectors such as LV and RV may be associated with long-term risk of genotoxicity and potential life long correction; AAV have a lower risk regarding integration, but are limited by cloning capacity and potential adaptive immune response to viral antigens. HDAd are associated with a dose-related innate immune response but offers efficient transduction without risk of genome integration. None of these obstacles are conceptually immovable and novel strategies need to be investigated to improve the safety profile of these vectors. Based on the significant progress to date, in spite of the expected setbacks of all drug development efforts, gene therapy for liver metabolic disorders may soon become a clinical reality.

## Competing interests

The author declares that she has no competing interests.
